# Supplementary material for: Molecular Phylogeny and Evolution of Amazon Parrots in the Greater Antilles
Source: Genes (Basel). 2021 Apr 20;12(4):608. doi: 10.3390/genes12040608 (PMC8074781; doi:10.3390/genes12040608)
Supplement: Supplementary file 1 [file genes-12-00608-s001.zip › genes-1159582-supplementary.pdf]

Supplementary Materials

Table S1. Origins of the *Amazona* spp. samples used in this study.

Species	n	Sample origin	Sample type	Sequencing method
Amazona vittata	10	Rio Abajo State Forest, Puerto Rico	blood, museum sample	Illumina[$], PacBio, Ion-Torrent[#], Sanger
A. ventralis	2	Rio Abajo State Forest, Puerto Rico	blood	Illumina
A. agilis	3	Hope Botanical Gardens and Zoo	blood	Illumina
A. collaria	7	Hope Botanical Gardens and Zoo	blood	Illumina
A. leucocephala	1	Frozen Zoo Collection, San Diego Zoo	skin graft	Illumina
A. albifrons	1	Aviculturist society donation (pet)	plucked feather	Illumina
A. rhodocorytha	1	Aviculturist society donation (pet)	plucked feather	Illumina

[$] See sequencing methods; [#] Not included in this study.

Table S2. Sets of overlapping primers for amplification of the full-length mitogenome of *A. vittata* and optimized melting temperatures used for annealing for amplification in this species. These primer pairs successfully amplified mtDNA for all the species in this study, except for the repeated control region reported by Urantowka *et al.* (2013).

Primer Pair ID	Primer IDs	Sequences	Tm #	T used	Product size
AE	L4500	GTAKCACAAACYATCTCCTAYGAAG	62	TD	2,200
	H6681	GGTAAAGGGTGCCGATGTCTTTGTG	72		
R	L13710	GGATCCTCHGCACTATCCATCCT	68	72	3,100
	H16191	TCTCGTGGGRCTATTCGGGC	71		
C	L3827	GAGYAATCCAGGTCGGTTTCTATC	66	66	2,100
	H5766	GGATGAGAAGGCTATRATTTTTCG	64		
E	L6335	GCCTTCAAAGCCTTAAACAAGAG	64	68	1,700
	H7956	GGGTAGTCCGAGTATCGTCG	64		
I	L7156	ACAGCCATCAACATAAAACCACC	66	72	1,500
	H8628	TCGTADGATCAGTATCATTGGTGTCC	67		
P	L12667	GTCTCCGCCCTACTCCACTCAAG	69	72	2,300
	H14790	GGGTGCTCTACTGGTTGGCTTC	68		
AM	L538	CCTCTGGTTCCTARGTCAGG	62	68	1,200
	H1858	TCGATTATAGAACAGGCTCCTCTAG	62		
AS	L538	CCTCTGGTTCCTARGTCAGG	62	66	1,100
	R2_DUP	AACGGTAAGGTTAGGACTAAGTC	59		
AZ	L9900	GGACTAATCATATGATTCCACT	57	TD	2,100
	H9549	GTCTATTGTAATGGATATACTAG	49		
K	L8316	TAACAATCAACCTCCTAGGCCT	63	68	1,700
	H9986	TGTTGAGTCGAAATCAACTGTC	62		
J	L7020	GGAAGGAATCGAACCCTCATA	64	62	2,000
	H10000	GTACAATGTCTCGTCATCATTG	60		
Ai	L9052	TCTGAGCCTTCTTCCACTCCAG	67	66	2,500
	H12488	ATACGGCTGTGTGTRCGTTC	64		
AQ	F2-AJ	ATTGACCTAAAATCACTCATC	55	58	700
	R2-AJ	TAGAGTTGTTTCCACTACTTTTAC	56		
Y	L12976	CAAGAACTGCTAAYTCTTGCATCTG	65	68	2,000
	H14500	TTTCGTAGGTTGGGGGCCATTAG	70		
AB	L1753	AAACTGGGATTAGATACCCCACTAT	63	66	2,300
	H4017	GCTAGRGAGAGGATTTGAACCTC	64		
AD	L3652	CCAGGGATAACAGCGCAATCTC	69	66	2,100
	H5766	GGATGAGAAGGCTATRATTTTTCG	64		
RR	L13710	GGATCCTCHGCACTATCCATCCT	68	68	500
	H1858	TCGATTATAGAACAGGCTCCTCTAG	62		

Tm – melting temperature; **TD* – Touch Down protocol 60–50°C

Table S3. Sequencing read outputs of Illumina paired-end libraries for the species used in this study.

Species	Insert size	Original		Filtered	
		**Total Reads** (millions)	**Total Bases** (Gb)	**Total Reads** (millions)	**Total Bases** (Gb)
Amazona vittata	200 bp	267	26	202	20
	3 kbp	153	15	83	8
	5 kbp	51	5	28	3
	8 kbp	385	38	283	29
A. leucocephala	300 bp	387	39	323	33
	3 kbp	89	8	49	4
A. ventralis	300 bp	408	49	378	32
	3 kbp	88	13	100	9
A. albifrons	400 bp	154	16	120	12
A. rhodocorytha	400 bp	149	15	115	11
A. agilis	400 bp	396	110	302	84
A. collaria	400 bp	379	106	298	82

Table S4. Sources of mitochondrial DNA sequences from additional parrot taxa used in this paper.

Species	NCBI ID	Source paper
Amazona aestiva	KT361659	Lima *et al.*, 2018
A. ochrocephala	KM611467	Eberhard and Wright, 2016
A. barbadensis	JX524615	Urantowka, Hajduk and Kosowska, 2013
Pionus chalcopterus	MF784450	Urantówka, Kroczak and Mackiewicz, 2017a
P. menstruus	KX925978	Urantówka and Mackiewicz, 2016
Ara militaris	KM611466	Eberhard and Wright, 2016
A. ararauna	KF010315	Urantowka, Mackiewicz and Strzała, 2017
A. severus	KF946546	Urantówka, Kroczak and Mackiewicz, 2017b
Aratinga mitrata	JX215256	Urantowka, Mackiewicz and Strzała, 2015
A. rubritorquis	JX524614	Urantówka, Kroczak and Strzała, 2013

Table S5. Parameters for the various models of biogeography used in the *BioGeoBEARS* analysis. The models include a likelihood version of DIVALIKE, DEC, and BAYAREALIKE, as well as "+J" versions of these three models which include founder-event speciation. .

**Model**	**DIVALIKE (A)**	**DIVALIKE +J (B)**	**DIVALIKE2 (C)**	**DI-VALIKE+J2(D)**	**DEC (E)**	**DEC +J (F)**	**BAYARELIKE (G)**	**BAYARELIKE+J (H)**
**Sympatry (*y*)**	*no*	*no*	*no*	*no*	*no*	*no*	*no*	*no*
**Vicariance (*v*)**	*yes*	*yes*	*yes*	*yes*	*default*	*default*	*no*	*no*
**Dispersal (*d,x*)**	*yes*	*yes*	*yes*	*yes*	*yes*	*yes*	*yes*	*yes*
**Extinction (*e*)**	*yes*	*yes*	*no*	*no*	*default*	*yes*	*default*	*yes*
**Jump dispersal (*j*)**	*no*	*yes*	*no*	*yes*	*no*	*yes*	*no*	*yes*

Table S6. The estimated sizes of the landmasses and matrix of shortest pairwise distances between locations in km. The upper part of the table corresponds to the time period starting from 3.3 MYA; bottom part – the period starting from 5 MYA. M - mainland; Y – Yucatan, C – Cuba, J – Jamaica, H – Hispaniola, P – Puerto Rico. This table has modified from the dist_matrix.txt input file for the BioGeoBEARS analysis.

	**Mainland (M)** #	**Yucatan (Y)** #	**Cuba (C)**	**Jamaica (J)**	**Hispaniola (H)**	**Puerto Rico (P)**
**Island Size (km^2)**	521,876	181,000	109,884	10,991	76,192	9,104
			**First time period (before 3.3 MYA)**			
	**M**	**Y**	**C**	**J**	**H**	**P**
**M**	-	1	650	**150**	**473**	**1,286**
**Y**		-	70	900	1,320	2,000
**C**			-	100	100	634
**J**				-	85	943
**H**					-	50
**P**						-
			**Second time period (3.3 MYA-present)**			
	**M**	**Y**	**C**	**J**	**H**	**P**
**M**	-	1	650	**650**	**473**	**1,286**
**Y**		-	100	900	1,320	2,000
**C**			-	100	86	750
**J**				-	100	950
**H**					-	60
**P**						-

The landmasses of the Central American Mainland and Yucatan are approximate and do not overlap (from R. W. McColl ed. (2005) Encyclopedia Of World Geography. Facts on File Library of World Geography, NY. 1216 p.)

Table S7. Node ages and 95% HPD age ranges from **Figure S5**.

**Node #**	**Height 95% HPD (Mya)**		**Height Median (Mya)**
	**min**	**max**	
**1**	18.71	32.31	25.35
**2**	6.88	11.95	9.31
**3**	4.42	7.71	6.00
**4**	2.52	4.46	3.47
**5**	2.29	4.03	3.14
**6**	1.01	1.81	1.39
**7**	0.55	1.01	0.77
**8**	0.49	0.9	0.69
**9**	3.48	6.12	4.76
**10**	0.88	1.58	1.21
**11**	0.26	0.51	0.38
**12**	2.3	4.1	3.20
**13**	3.6	6.34	4.96
**14**	3.8	6.65	5.19
**15**	7.41	12.83	10.06
**16**	1.18	2.13	1.64

Table S8. Absolute character differences within four species of Greater Antillean *Amazona* parrots.

Species	Total differences	Coding regions	Non-coding regions
A. vittata	34	0	34
A. ventralis	114	18	96
A. collaria	97	13	84
A. agilis	112	0	112

Table S9. Pairwise absolute character differences among five species of Greater Antillean *Amazona* parrots based on alignment to *A. agilis*.

Species	Total differences	Coding regions	Non-coding regions
A. vittata	998	696	302
A. ventralis	999	698	301
A. leucocephala	991	677	314
A. collaria	988	668	320
A. albifrons	1011	730	281

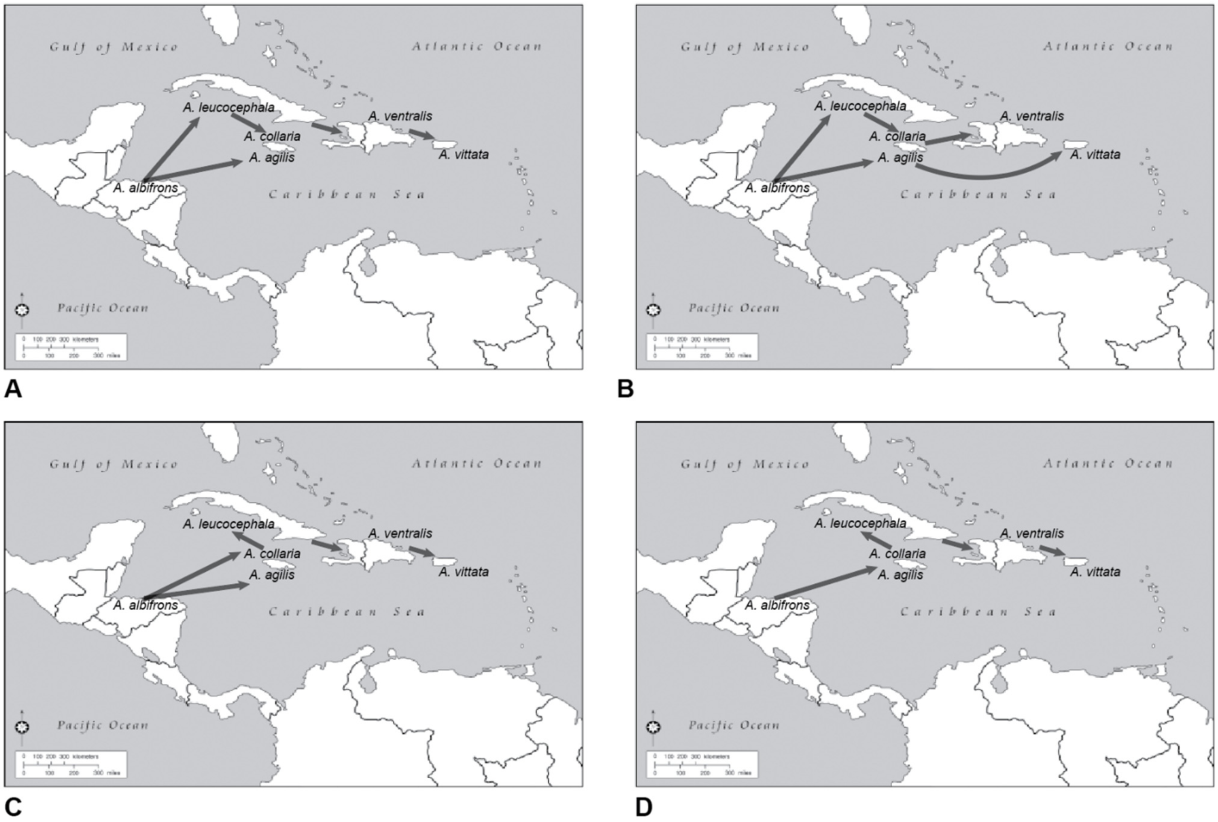

Figure S1. Alternative dispersal-speciation scenarios have been proposed for the evolution of Greater Antillean island *Amazona* parrots by overwater dispersal from Central America (A, B - modified after Russello and Amato, 2004). In the model **A** [21], the ancestors dispersed from Central America to Cuba, Hispaniola and Puerto Rico, using each island as a stepping stone before reaching the next one. Jamaican species are the result of two independent dispersal events, one from Cuba and one directly from Central America. In the model **B** [15], Jamaica is proposed as a stepping stone for the further dispersals to Hispaniola and Puerto Rico. Model **C** (Ottens-Wainright 2004; Russello & Amato 2004) is different from A in that ancestors of A. collaria first arrive to Jamaica and only then give rise to the subpopulation which goes to Cuba and becomes the founder population of A. leucocephala. High-resolution image here: https://drive.google.com/file/d/1BnY-TxgSJ57ZNiTl9g8wVljMftT4WABvV/view?usp=sharing.

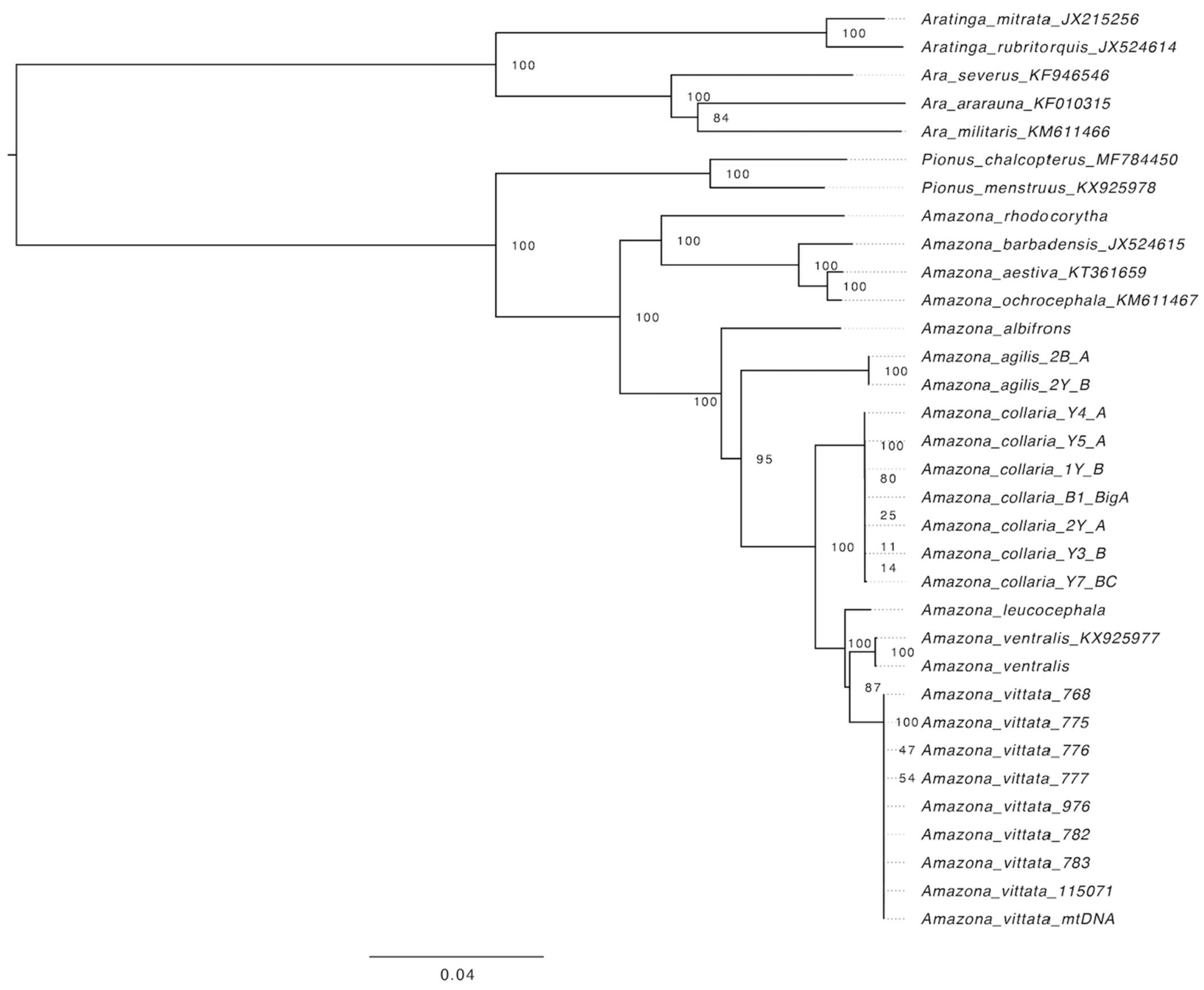

Figure S2. Maximum likelihood tree for the Greater Antillean amazons in the larger context of Neotropical parrot evolution, reconstructed with IQ-TREE 1.6.9 [50]. The tree topology is congruent with the one obtained using Bayesian methods (see Figure 6), which supports the most likely phylogenetic relationships between the species of interest: *A. vittata* and *A. ventralis* are sister species, *A. leucocephala* is basal to both of them. Among the Jamaican parrots an earlier split is observed for *A. agilis*, which is basal to all the other Greater Antillean amazons, and *A. collaria* is more closely related to *A. leucocephala*, than to *A. agilis.* Bootstrap percentages (1000 replicates) are shown at internal nodes. High-resolution image here: https://drive.google.com/file/d/1w56XNIQNGq4OgluUOERDzb9tpHU2_kvq/view?usp=sharing

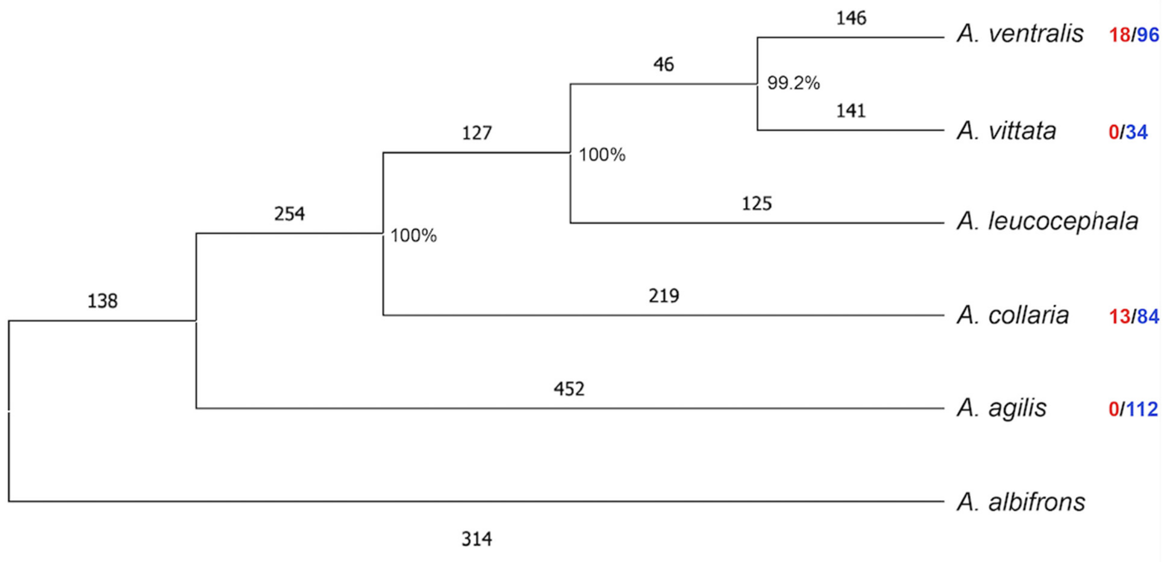

Figure S3. The maximum parsimony (MP) tree depicting absolute numbers of nucleotide changes relative to the common ancestor along phylogenetic lineages (indicated next to each branch) and intraspecific diversity in species with multiple individuals (numbers to the right: coding differences indicated in red, non-coding – in blue). Bootstrap percentages (500 replicates) are shown at internal nodes. High-resolution image here: https://drive.google.com/file/d/1L5FmZdECKyy9k1tBGf-cXb2wrWl03gtN/view?usp=sharing.

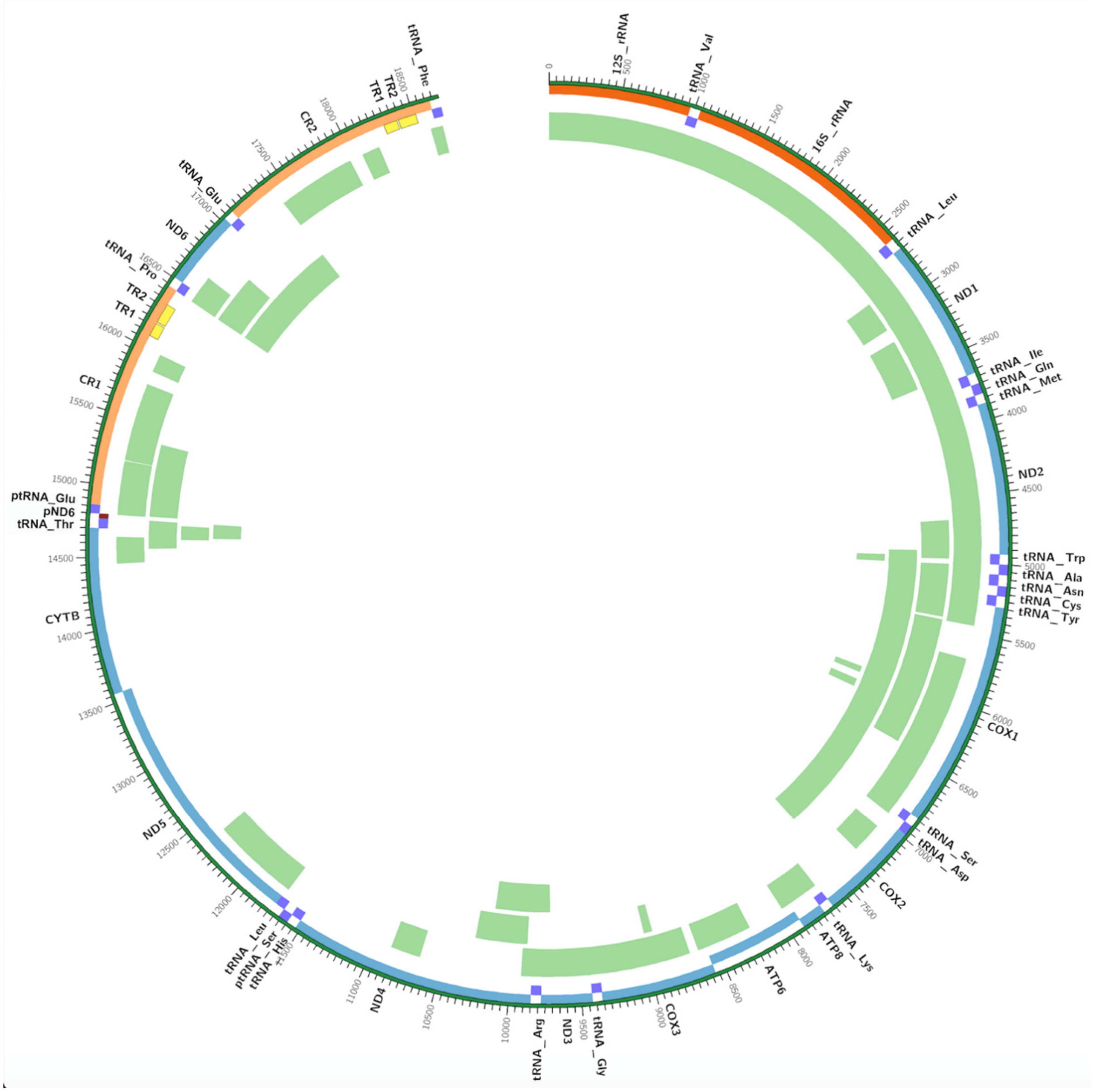

Figure S4. Candidate nuclear copies of mitochondrial DNA (NUMTs) with e-values equal to or smaller than 10^{-4} found in the most recent nuclear *Amazona vitatta* genome assembly (Kolchanova *et al.* 2019) aligned to the *A. vittata* mtDNA (green bars). Detailed information about the 34 discovered candidate NUMTs can be found in **Appendix C**. High-resolution here: https://drive.google.com/file/d/1RB0rYGMis7rYMryHHxJ-NN_6O7sM7gqM/view?usp=sharing.

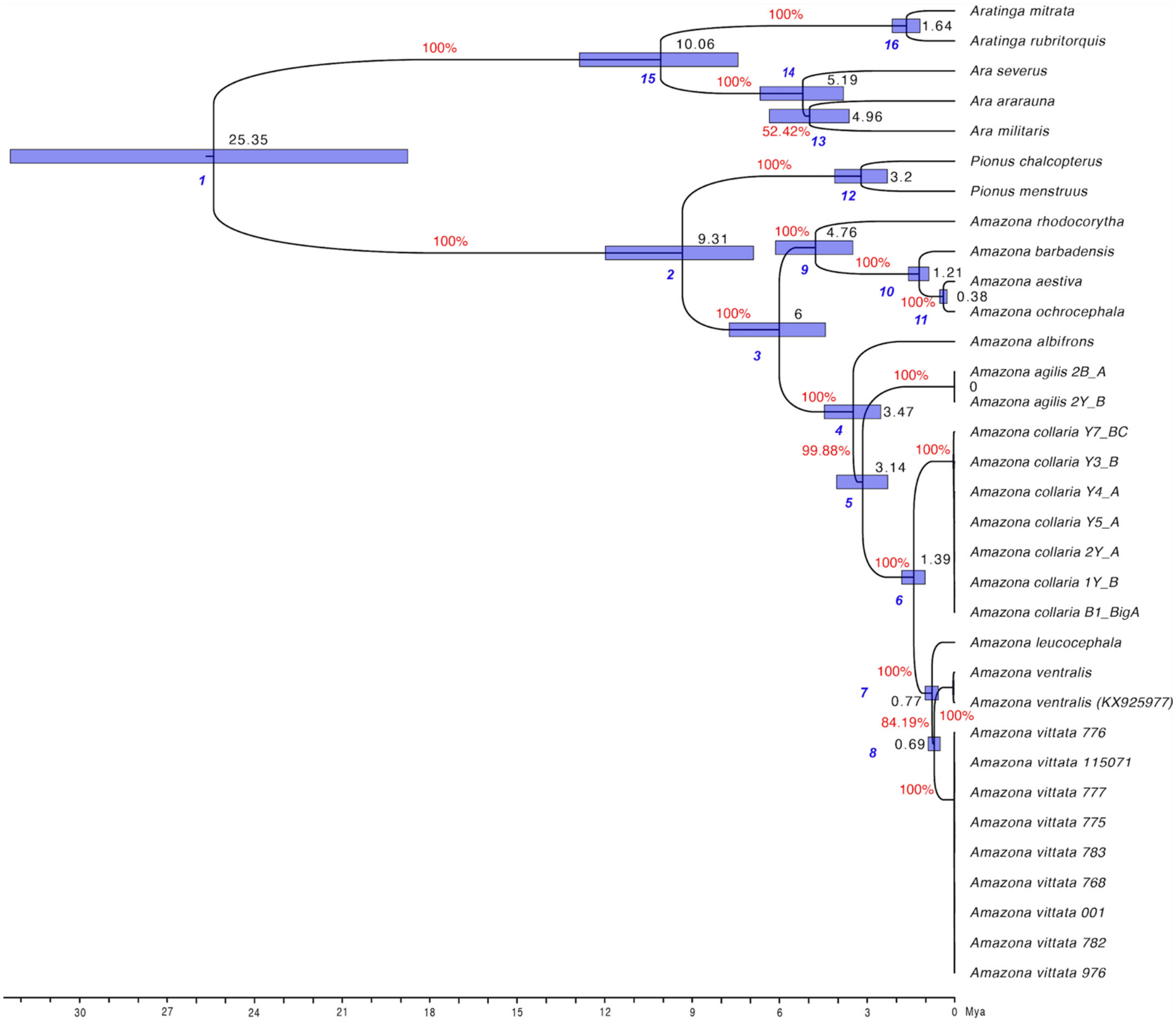

Figure S5. Full phylogeny acquired as a result of the BEAST analysis using strict clock. Blue numbers in bold indicate the node number, as listed in Table S5. Red numbers are branch support, black numbers are node ages, blue bars are confidence intervals for node ages. High-resolution here: https://drive.google.com/file/d/13X-G-f0PRNCsziRW6yANwZx5OdYuqlDo/view?usp=sharing.

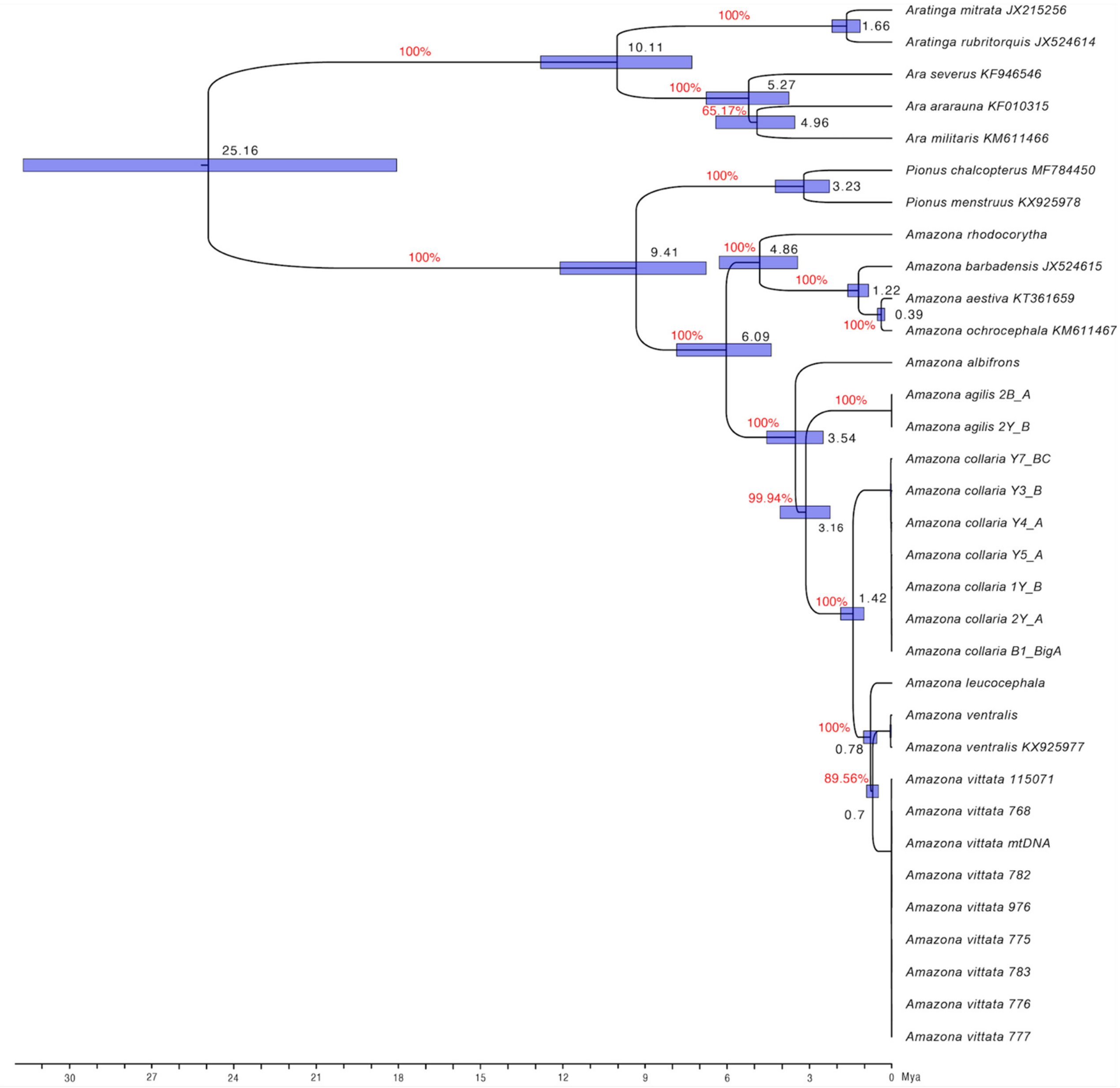

Figure S6. Full phylogeny acquired as a result of the BEAST analysis using a relaxed clock with evolutionary rates at each branch drawn from a log-normal probability distribution. Red numbers are branch support, black numbers are node ages, blue bars are node age confidence intervals. High-resolution here: [https://drive.google.com/file/d/1MjHZnWz_zEWA-Yi5B8tBdQJ2-CF9QG2Q/view?usp=sharing.](https://drive.google.com/file/d/1MjHZnWz_zEWA-Yi5B8tBdQJ2-CF9QG2Q/view?usp=sharing)

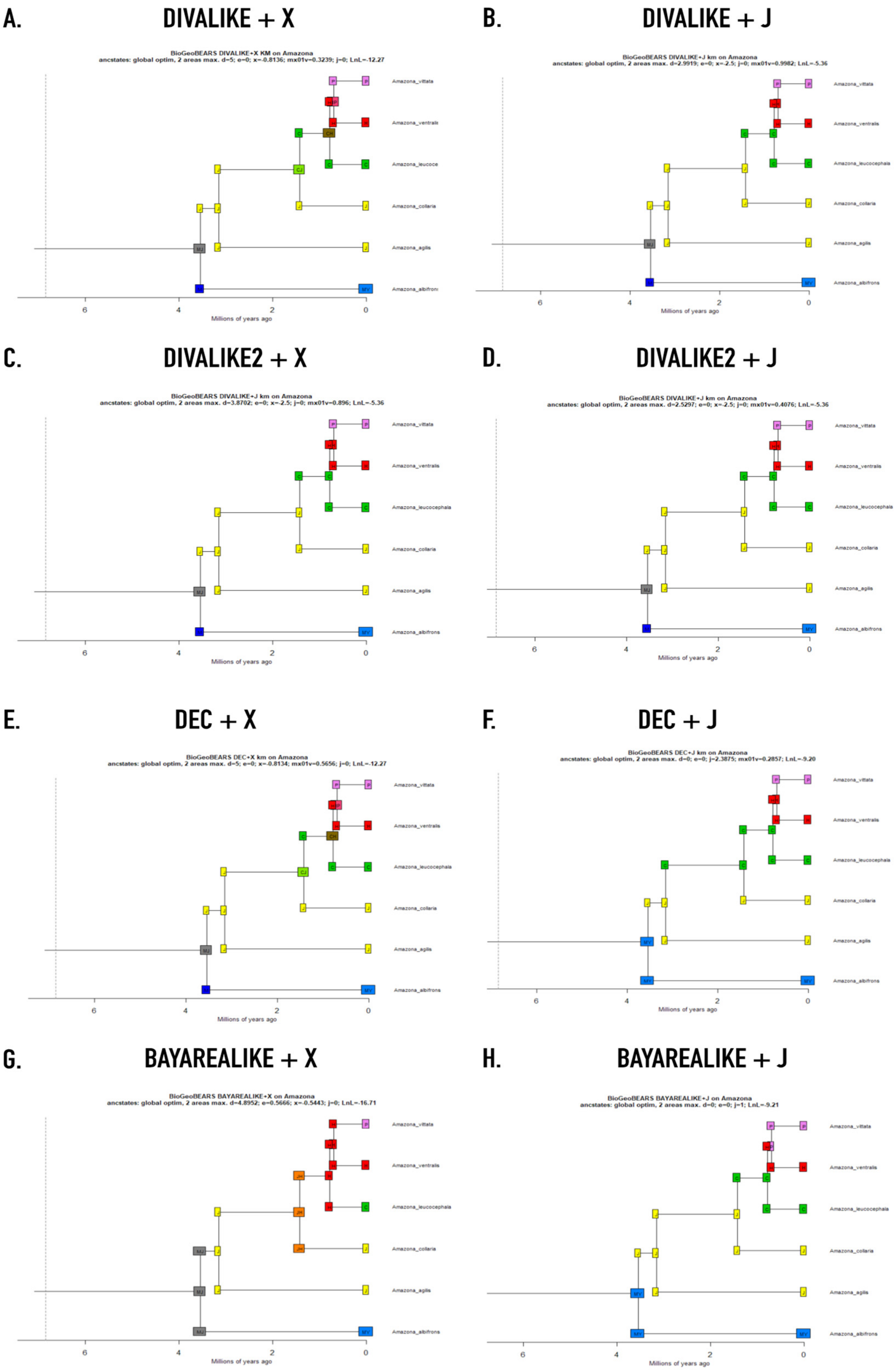

Figure S7. Speciation patterns predicted by different biogeographical models tested to infer possible speciation and dispersal routes using the *BioGeoBEARS* package [63] using a distance matrix based on the shortest distances between the islands (**Table S6**).

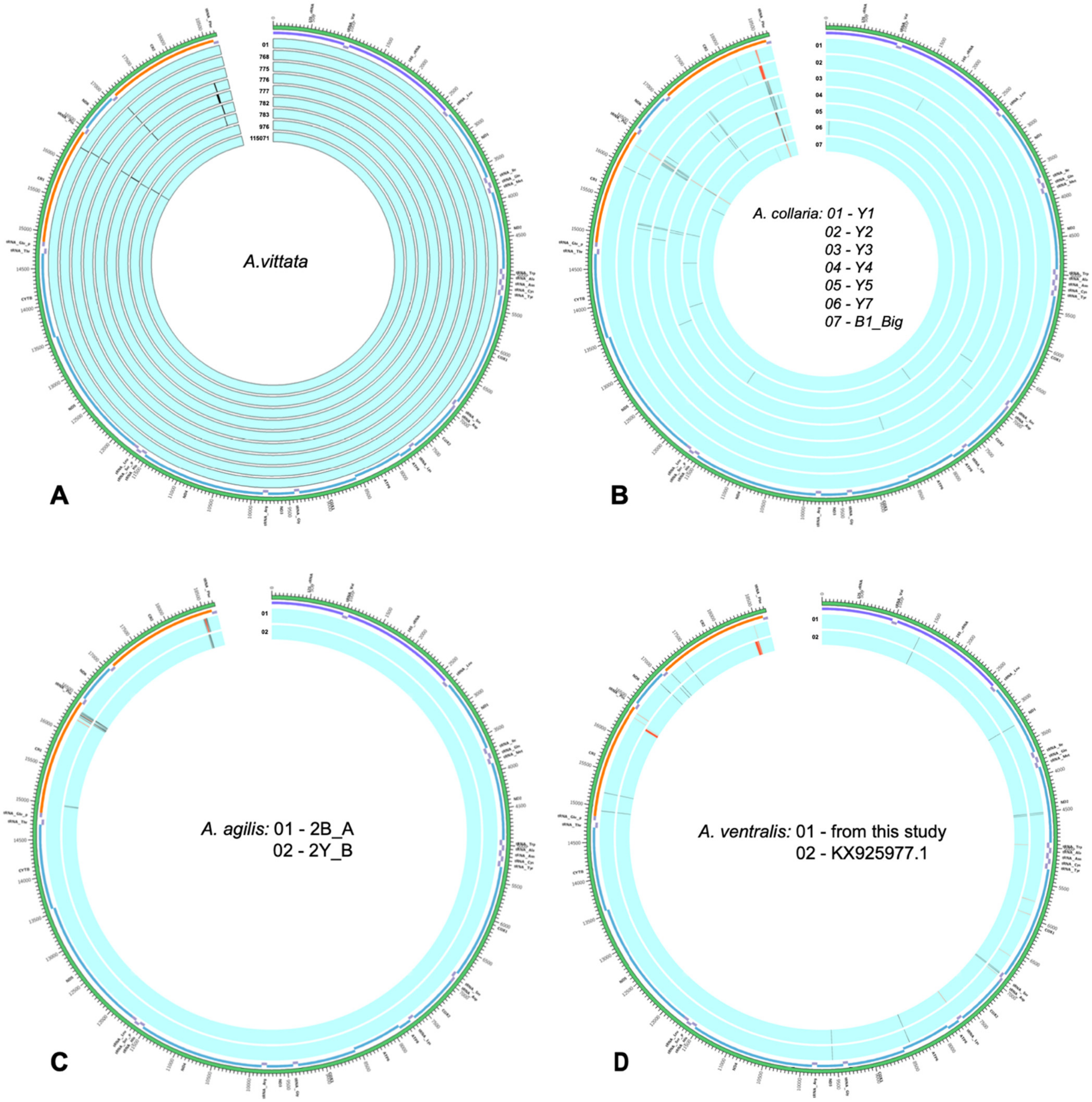

Figure S8. Locations of variable sites in four intraspecific alignments. (A) – *Amazona vittata*, n = 9; (B) *Amazona collaria*, n = 7; (C) *Amazona agilis*, n = 2; (D) *Amazona ventralis*, n = 2. Black bars represent base substitutions, red bars – indels. High resolution here: https://drive.google.com/file/d/1knqSdbWkJct7pncn8egjgj6UVZ0N7A-T/view?usp=sharing.
